# Antioxidant Intervention in NAFLD: Astaxanthin and Kokum Modulate Redox Status and Lysosomal Degradation

**DOI:** 10.3390/molecules31020321

**Published:** 2026-01-16

**Authors:** Natalia Ksepka, Natalia Kuzia, Sara Frazzini, Luciana Rossi, Małgorzata Łysek-Gładysińska, Michał Ławiński, Artur Jóźwik

**Affiliations:** 1 Department of Biotechnology and Nutrigenomics, Institute of Genetics and Animal Biotechnology, Polish Academy of Sciences, Postępu 36a, Jastrzębiec, 05-552 Magdalenka, Poland; n.kuzia@igbzpan.pl (N.K.); aa.jozwik@igbzpan.pl (A.J.); 2 Department of Veterinary Medicine and Animal Sciences-DIVAS, University of Milan, Via dell’Università 6, 26900 Lodi, Italy; sara.frazzini@unimi.it (S.F.); luciana.rossi@unimi.it (L.R.); 3 Division of Medical Biology, Institute of Biology, University of Jan Kochanowski, 25-406 Kielce, Poland; malgorzata.lysek-gladysinska@ujk.edu.pl; 4 Department of General, Gastroenterology, and Oncologic Surgery, Medical University of Warsaw, Banacha 1a, 02-097 Warsaw, Poland

**Keywords:** NAFLD, oxidative stress, lysosomes, gene expression, astaxanthin, kokum

## Abstract

Non-alcoholic fatty liver disease (NAFLD) is a major metabolic disorder characterized by hepatic lipid accumulation, oxidative stress, and disturbance of lysosomal degradation. Central to these processes is glutathione (GSH), a key antioxidant regulating redox balance and cellular homeostasis. This study aimed to evaluate the therapeutic potential of two dietary antioxidants—astaxanthin and *Garcinia indica* (kokum)—in modulating hepatic redox status, lysosomal function, and metabolic gene expression in a murine model of diet-induced NAFLD. A total of 120 male Swiss Webster mice were allocated into control and steatotic groups, followed by a 90-day supplementation period with astaxanthin, kokum, or their combination. Liver tissue was collected post-supplementation for biochemical, antioxidant, and qRT-PCR analyses. Outcomes included lysosomal enzymes activities, superoxide dismutase (SOD), GSH, vitamin C, total polyphenols, DPPH radical-scavenging activity, and total antioxidant capacity (TAC). NAFLD induced marked oxidative stress, lysosomal overactivation, and alteration of antioxidant-related gene expression. Combined supplementation restored GSH, enhanced TAC, reduced lysosomal stress markers, and significantly upregulated nuclear factor erythroid 2-related factor 2 (Nfe2l2) while downregulating fatty acid synthase (FASN) and partially rescuing lipoprotein lipase (LpL). Correlation analyses revealed strong associations between antioxidant capacity, lysosomal function, and transcriptional regulation, supporting the therapeutic relevance of combined antioxidant therapy for concurrent redox and lysosomal dysregulation in NAFLD. These findings underscore the therapeutic potential of targeting redox and cellular degradation pathways with antioxidant-based interventions to re-establish hepatic metabolic balance in NAFLD and related disorders.

## 1. Introduction

Non-alcoholic fatty liver disease (NAFLD) is the most prevalent chronic liver disorder worldwide, affecting approximately 25–30% of the population. Its incidence continues to rise in parallel with the growing epidemic of metabolic syndrome [1]. NAFLD encompasses a spectrum of pathological conditions, beginning with simple hepatic steatosis (NAFL) and potentially progressing to non-alcoholic steatohepatitis (NASH), fibrosis, cirrhosis, and ultimately hepatocellular carcinoma [2]. NAFLD develops through multifactorial mechanisms and has evolved from the earlier “two-hit” hypothesis to the more comprehensive “multiple-hit” model, which incorporates insulin resistance, gut microbiota dysbiosis, genetic predisposition, and—most prominently—oxidative stress [1,3].

Oxidative stress, defined by an imbalance between reactive oxygen species (ROS) production and antioxidant defenses, plays a pivotal role in the transition from simple steatosis to NASH [4,5]. Hepatic lipid overload induces lipotoxicity, mitochondrial dysfunction, and endoplasmic reticulum (ER) stress, leading to increased ROS generation, lipid peroxidation, DNA damage, and activation of redox-sensitive inflammatory signaling pathways [6,7]. These events further promote hepatocellular injury, activation of Kupffer cells and hepatic stellate cells, and progression to fibrosis [8,9].

It is noteworthy that the balance between oxidative processes and antioxidant defense can be disrupted not only by excessive reactive oxygen species production but also through overloading with exogenous antioxidants. Under such conditions, excessive antioxidant availability may interfere with physiological redox signaling, making it challenging to avoid possible toxic effects [10,11].

In response to oxidative stress, the antioxidant system is upregulated, encompassing both enzymatic and non-enzymatic components. Among the enzymatic antioxidants, superoxide dismutase (SOD) plays significant role by catalyzing the dismutation of superoxide radicals (O_2_^−^) into hydrogen peroxide and molecular oxygen. Three SOD isoforms are found in mammals: cytosolic SOD1, mitochondrial SOD2, and extracellular SOD3 (EC-SOD). Studies have shown decreased SOD expression, particularly EC-SOD, in NAFLD, and this exacerbates oxidative damage and inflammation [12,13].

Glutathione (GSH) is a major non-enzymatic antioxidant and essential for maintaining redox homeostasis in hepatocytes by detoxifying ROS, preserving mitochondrial integrity, and modulating cell—death pathways [14,15]. In NAFLD, depletion of GSH and downregulation of GSH-dependent enzymes such as glutathione peroxidase (GPx) and glutathione S-transferase (GST) contribute to hepatic oxidative damage [3,4,16]. Accordingly, therapeutic strategies aimed at restoring GSH have shown promise in mitigating oxidative stress and improving liver function in preclinical models [17,18,19].

Complementary to the GSH system, total antioxidant capacity (TAC) reflects the combined effect of enzymatic and non-enzymatic antioxidants [20]. Clinical data suggest that TAC is significantly reduced in patients with NAFLD and negatively correlates with steatosis, inflammation, and fibrosis [9,21]. Dietary total antioxidant capacity (DTAC) is also inversely associated with NAFLD risk and severity, underscoring the importance of nutritional antioxidant support [22].

Further, the transcriptional pathways regulating antioxidant and metabolic responses include the activation of central energy sensor which influences lipid metabolism, oxidative stress, and lysosomal degradation. Fatty acid synthase (FASN), a key enzyme in de novo lipogenesis, catalyzes palmitate synthesis and is overexpressed in insulin resistance, promoting hepatic lipid accumulation in NAFLD [23,24]. Lipoprotein lipase (LpL) facilitates the hydrolysis of triglycerides from circulating lipoproteins, promoting fatty acid uptake by tissues [25]. Genetic studies indicate that enhanced LpL activity may reduce NAFLD risk independently of plasma lipid concentrations, highlighting a potential protective role in hepatic lipid handling [26].

Astaxanthin, a dark red xanthophyll carotenoid, occurs naturally in microorganisms and marine organisms, including prawns, crabs, salmon, and its primary source, the freshwater microalga *Haematococcus pluvialis*. In humans, dietary exposure to astaxanthin is generally low and variable; therefore, it is more commonly consumed as a dietary supplement. Astaxanthin exhibits potent antioxidant properties and has been shown to improve mitochondrial function, restore redox balance, and reduce hepatic steatosis in experimental models of NAFLD [2,27,28]. *Garcinia indica* (kokum) is an evergreen tropical tree native to parts of India. Its fruit contains of garcinol, a crystalline polyisoprenylated benzophenone belonging to the polyphenol family, which exhibits antioxidant and anti-inflammatory activity. Although kokum fruit is traditionally consumed in regional Indian diets, its intake is geographically limited, and garcinol is most often studied as a component of concentrated extracts or supplements in experimental research [29,30,31,32,33,34]. Together, these compounds may complement each other by modulating mitochondrial function, lipid metabolism, and overall redox homeostasis, making them promising candidates for targeting oxidative stress-related pathways in NAFLD.

Understanding how nutraceutical antioxidants influence redox markers, lysosomal activity, and metabolic gene expression may provide future therapeutic strategies. The aim of the present study was to assess hepatic oxidative stress, antioxidant defenses, and lysosomal enzyme activity in a murine NAFLD model and to determine the hepatoprotective effects of astaxanthin and kokum, administered individually or in combination. In addition, we describe the molecular associations between oxidative stress and lysosomal dysfunction in NAFLD, thereby identifying potential therapeutic targets within the glutathione–lysosomal degradation axis.

## 2. Results

The statistical analysis was conducted to compare the impact of liver steatosis and different supplementation strategies on selected antioxidant parameters in mouse liver homogenates. The animals were divided into control groups and groups with diet-induced non-alcoholic fatty liver disease, each further subdivided based on supplementation with astaxanthin, kokum, or their combination.

**Figure 1:**
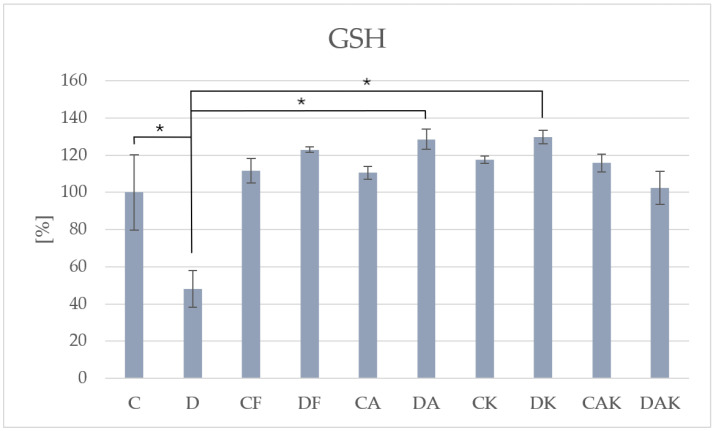
The level of reduced glutathione in mice liver; * *p* < 0.05; C—initial control group; D—hepatic steatosis group; CF—continued control group; DF—post-steatosis group; CA—control group supplemented with astaxanthin; DA—hepatic steatosis group supplemented with astaxanthin; CK—control group supplemented with kokum; DK—hepatic steatosis group supplemented with kokum; CAK—control group with combined supplementation; DAK—hepatic steatosis group with combined supplementation.

Analysis of reduced glutathione levels revealed a significantly lower concentration in group D (337.87 ± 33.09) compared to all control and supplemented groups (Table 1). Supplementation led to a marked increase in GSH levels, particularly in the DA (902.05 ± 49.12) and DK (911.43 ± 33.35) groups (Figure 1).

**Figure 2:**
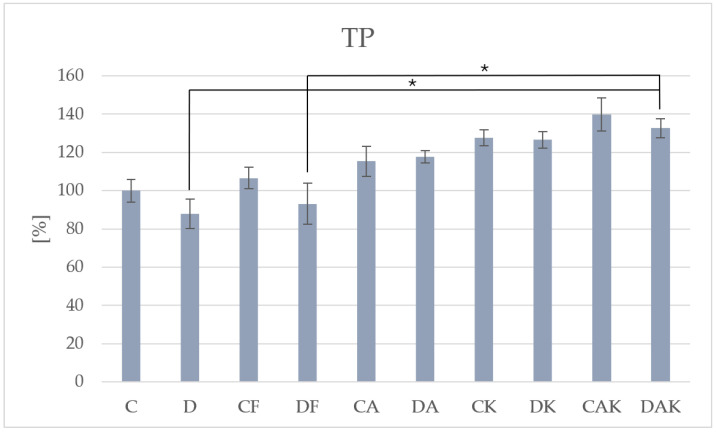
The level of total polyphenols in mice liver; * *p* < 0.05; C—initial control group; D—hepatic steatosis group; CF—continued control group; DF—post-steatosis group; CA—control group supplemented with astaxanthin; DA—hepatic steatosis group supplemented with astaxanthin; CK—control group supplemented with kokum; DK—hepatic steatosis group supplemented with kokum; CAK—control group with combined supplementation; DAK—hepatic steatosis group with combined supplementation.

In NAFLD mice, the highest total polyphenol content was observed in the synergistically supplemented group—DAK (7.52 ± 0.37)—which exhibited significantly higher levels compared to groups D (4.99 ± 0.39) and DF (5.28 ± 0.56) (Figure 2). These results confirm an enhanced antioxidant capacity in response to nutraceutical intake.

The DPPH radical scavenging activity was highest in group D (78.45 ± 4.38) (Table 1), indicating elevated oxidative stress under steatotic conditions. Supplementation with antioxidants effectively reduced DPPH values in all groups. This reduction reflects the potential of antioxidant supplementation to mitigate oxidative stress, especially in the context of liver damage.

Vitamin C levels were significantly lower in group D (2.68 ± 0.63) compared to group C (5.19 ± 0.37) (Table 1). Although supplementation improved vitamin C concentrations, it did not fully normalize them, suggesting an increased utilization of ascorbic acid during liver regeneration.

**Figure 3:**
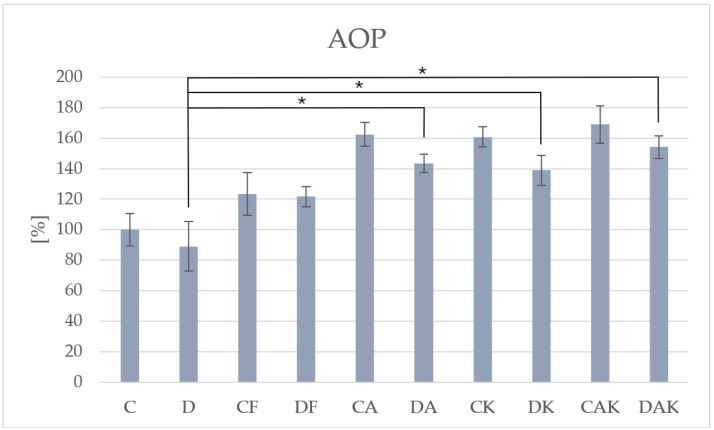
The level of total antioxidant potential in mice liver; * *p* < 0.05; C—initial control group; D—hepatic steatosis group; CF—continued control group; DF—post-steatosis group; CA—control group supplemented with astaxanthin; DA—hepatic steatosis group supplemented with astaxanthin; CK—control group supplemented with kokum; DK—hepatic steatosis group supplemented with kokum; CAK—control group with combined supplementation; DAK—hepatic steatosis group with combined supplementation.

The lowest AOP was recorded in group D (4.30 ± 0.70) (Table 1). However, it increased significantly in all supplemented groups (Figure 3), further confirming the beneficial effect of supplementation on redox homeostasis.

This hypothesis is further supported by the activity of superoxide dismutase, which also increased significantly in all supplemented groups compared to group D (2.60 ± 0.69) (Table 1). These findings indicate the activation of endogenous protective mechanisms in response to antioxidant intervention.

Dietary supplementation with astaxanthin and kokum, particularly when administered in combination (CAK, DAK), contributed to the restoration of hepatic cellular homeostasis by modulating multiple degradation-associated biochemical parameters. These findings support the high antioxidant potential of the natural compounds used, effectively mitigating oxidative stress associated with liver regeneration.

**Figure 4:**
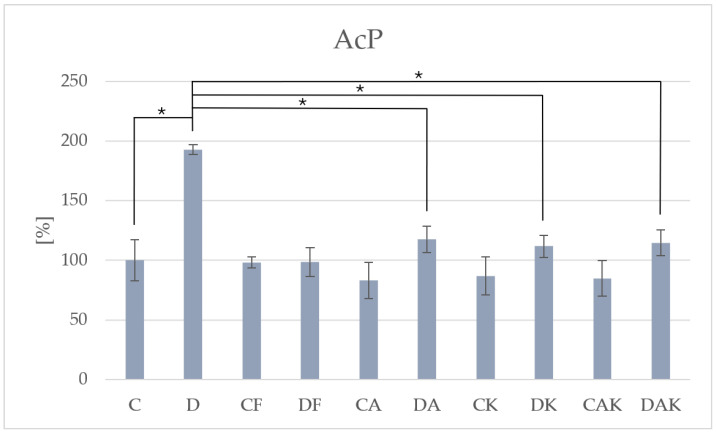
The activity of acid phosphatase in mice liver; * *p* < 0.05; C—initial control group; D—hepatic steatosis group; CF—continued control group; DF—post-steatosis group; CA—control group supplemented with astaxanthin; DA—hepatic steatosis group supplemented with astaxanthin; CK—control group supplemented with kokum; DK—hepatic steatosis group supplemented with kokum; CAK—control group with combined supplementation; DAK—hepatic steatosis group with combined supplementation.

The key lysosomal degradation marker, acid phosphatase, exhibited significantly elevated activity in group D prior to antioxidant intervention (3002.79 ± 127.90), compared to group C (1556.65 ± 269.54), indicating intensified lysosomal activity due to hepatic injury (Table 2). Dietary antioxidant intervention resulted in marked inhibition of AcP activity, suggesting a shift towards hepatic repair and restoration of lysosomal balance (Figure 4).

**Figure 5:**
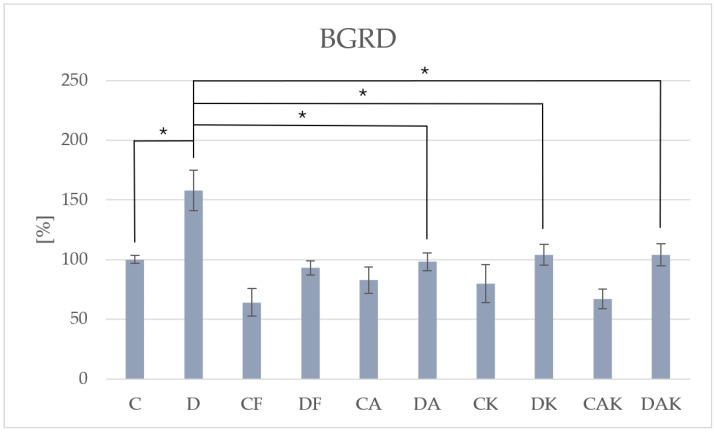
The activity of β-glucuronidase in mice liver; * *p* < 0.05; C—initial control group; D—hepatic steatosis group; CF—continued control group; DF—post-steatosis group; CA—control group supplemented with astaxanthin; DA—hepatic steatosis group supplemented with astaxanthin; CK—control group supplemented with kokum; DK—hepatic steatosis group supplemented with kokum; CAK—control group with combined supplementation; DAK—hepatic steatosis group with combined supplementation.

The presence of steatosis was further confirmed by increased activity of β-glucuronidase in group D (292.49 ± 48.99) (Figure 5), an enzyme involved in lipid detoxification during hepatic fat accumulation. Antioxidant treatment significantly reduced BGRD activity, indicating attenuation of lipid-induced lysosomal stress.

Elevated hexosaminidase activity observed in group D (808.45 ± 178.72) reflected disturbances in carbohydrate and lipid turnover under steatotic conditions. Notably, separate supplementation with kokum and astaxanthin led to a significant reduction in HEX activity in mice with steatosis (DK-601.44 ± 28.69; DA-613.26 ± 105.01) (Table 2).

**Figure 6:**
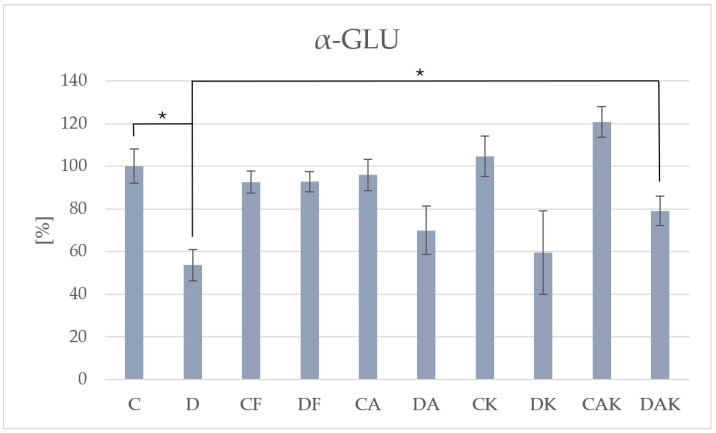
The activity of α-glucosidase in mice liver; * *p* < 0.05; C—initial control group; D—hepatic steatosis group; CF—continued control group; DF—post-steatosis group; CA—control group supplemented with astaxanthin; DA—hepatic steatosis group supplemented with astaxanthin; CK—control group supplemented with kokum; DK—hepatic steatosis group supplemented with kokum; CAK—control group with combined supplementation; DAK—hepatic steatosis group with combined supplementation. supplementation.

Activities of α-GLU and β-GLU were lowest in group D (131.33 ± 9.50 and 44.52 ± 1.98, respectively), consistent with impaired carbohydrate metabolism. Following antioxidant intervention, both astaxanthin and kokum supplementation increased the activity of these enzymes in steatotic groups. Notably, the most pronounced effect was observed for α-GLU in the DAK group (193.64 ± 13.43) (Figure 6), indicating a synergistic response to combined supplementation, whereas changes in β-GLU activity (54.10 ± 4.48) were less marked. This pattern may reflect reactivation of key enzymes involved in hepatic carbohydrate processing. The observed increase in enzyme activity in supplemented control groups suggests normalization of hepatic carbohydrate metabolism.

Reduced activity of β-GAL (Table 2) in supplemented group DAK (198.84 ± 28.05), compared to D group (234.05 ± 20.12), suggests a synergistic protective effect of astaxanthin and kokum. This may indicate reduced lysosomal degradation of glycoproteins and glycolipids, and preservation of membrane integrity.

Aminopeptidases, including AlaAP, ArgAP, and LeuAP are critical enzymes implicated in protein catabolism and immune regulation. Their hepatic activity is closely linked to physiological and pathological processes such as inflammation, tissue remodeling, and fibrogenesis. In the present study, substantial intergroup differences in aminopeptidase activities (Table 3) were observed, suggesting dynamic regulation of proteolytic processes in response to steatotic conditions and dietary interventions.

**Figure 7:**
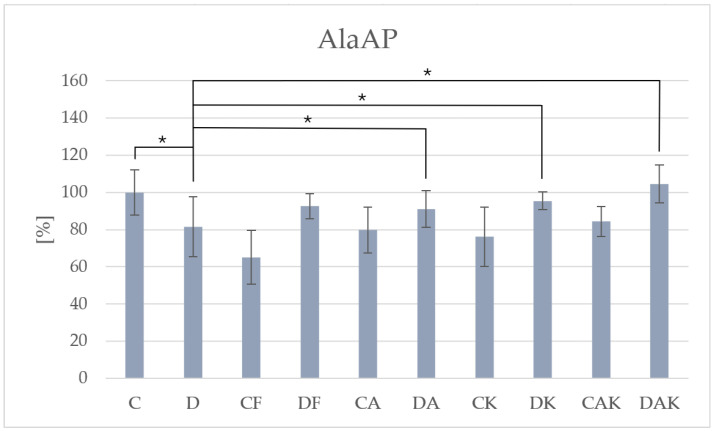
The activity of alanyl-aminopeptidase in mice liver; * *p* < 0.05; C—initial control group; D—hepatic steatosis group; CF—continued control group; DF—post-steatosis group; CA—control group supplemented with astaxanthin; DA—hepatic steatosis group supplemented with astaxanthin; CK—control group supplemented with kokum; DK—hepatic steatosis group supplemented with kokum; CAK—control group with combined supplementation; DAK—hepatic steatosis group with combined supplementation. supplementation.

In the case of AlaAP, enzymatic activity decreased significantly in group D (260.17 ± 42.17) compared to group C (318.91 ± 38.92). In all control groups, the trend of reduced alanyl-aminopeptidase activity persisted, whereas in the steatosis groups, discontinuation of the steatosis-inducing diet combined with nutraceutical supplementation resulted in an increase in activity compared to group D (Figure 7).

In the control groups, antioxidant intervention caused a decrease in ArgAP activity compared to group C (348.24 ± 13.36). Across the supplemented steatotic groups, activity increased, consistent with the AlaAP trend, with the maximal increase observed in the group treated with both components simultaneously (270.88 ± 47.68).

A significant decrease in LeuAP activity was observed in the non-steatotic groups supplemented with antioxidants compared to group C (421.51 ± 26.84). In the steatosis groups, however, the changes were generally not statistically significant compared to group D (395.90 ± 57.06), with the exception of DK (311.86 ± 14.15), which exhibited a significant decrease in LeuAP activity.

Importantly, the marked variability in aminopeptidase activities (Table 3) across the experimental groups is consistent with dysregulated protein catabolism and increased nitrogen turnover under steatotic conditions. These findings further underscore the presence of systemic metabolic disturbances in NAFLD, which appear to be partially normalized upon antioxidant supplementation.

**Figure 8:**
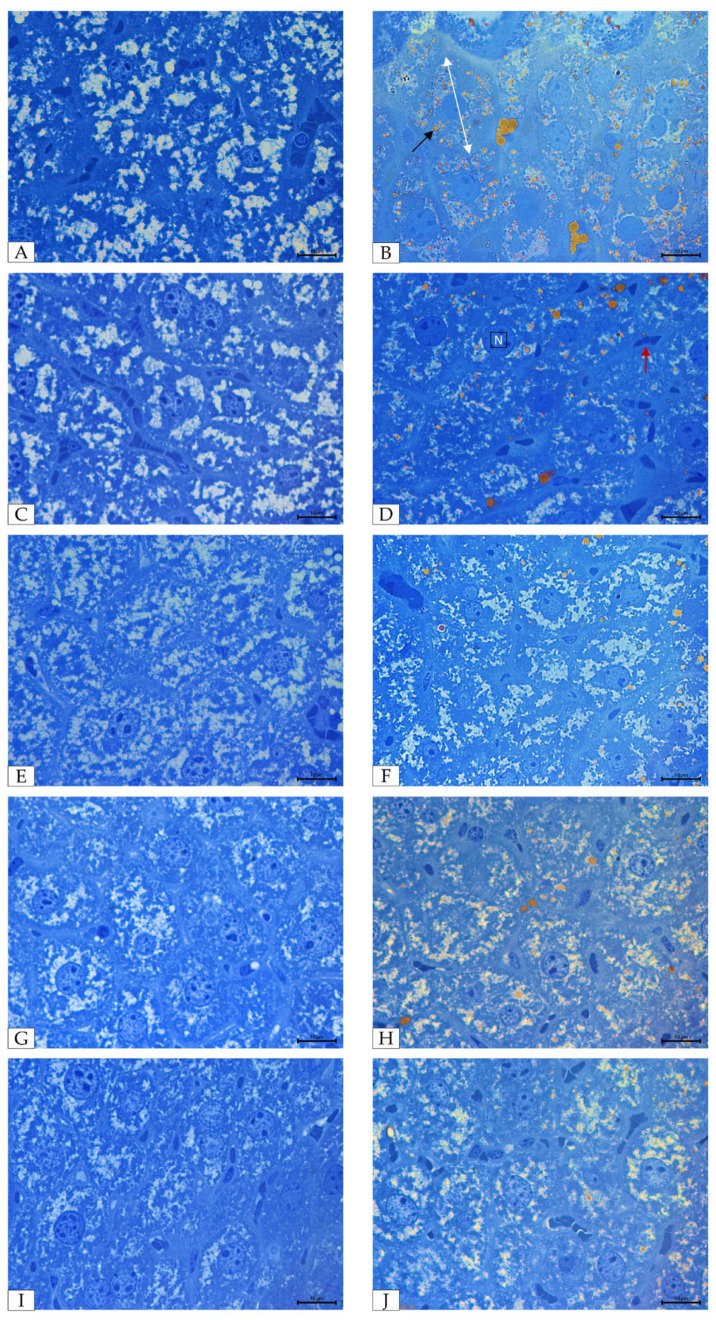
Microphotographs of mouse liver sections stained with Sudan III and aniline blue (×1000, light microscope). Panels (**A**,**C**,**E**,**G**,**I**) show representative liver sections from mice fed a standard diet: (**A**,**C**)—control; (**E**)—after astaxanthin supplementation; (**G**)—after kokum supplementation; (**I**)—after combined astaxanthin and kokum supplementation. Panels (**B**,**D**,**F**,**H**,**J**) show representative liver sections from mice induced NAFLD: (**B**,**D**)—without supplementation; (**F**)—after astaxanthin supplementation; (**H**)—after kokum supplementation; (**J**)—after combined astaxanthin and kokum supplementation. Black arrow indicates lipid droplets; white arrow indicates hepatocyte; red arrow indicates sinusoids; nucleus (N); Scale bars = 10 μm.

Histological evaluation of liver tissue stained with Sudan III and aniline blue revealed marked differences between the experimental groups (Figure 8). In the control mice fed a standard diet (Figure 8A,C), hepatocytes exhibited a normal polygonal shape with clearly defined cell borders, centrally located nuclei, and clear cytoplasm without lipid inclusions. Sinusoids were patent and non-dilated. In contrast, liver tissue from mice fed a MCD diet to induce NAFLD (Figure 8B) showed hepatocellular swelling, enlarged nuclei, and a substantial accumulation of lipid droplets within the cytoplasm compared with the control group. After returning to the standard diet (Figure 8D), hepatocytes displayed partial improvement, with a reduced number of lipid droplets and condensed nuclear chromatin, although sinusoidal dilatation was still visible. In the groups supplemented with astaxanthin (Figure 8F), kokum (Figure 8H), or their combination (Figure 8J), a pronounced reduction in lipid droplet accumulation was observed compared to group D. The combined treatment (Figure 8J) showed the most noticeable improvement, with hepatocytes maintaining a morphology similar to that of the control group. Mice fed a standard diet and supplemented with astaxanthin (Figure 8E), kokum (Figure 8G), or their combination (Figure 8I) did not exhibit significant morphological alterations relative to group C (Figure 8A,C).

To evaluate the molecular effects of hepatic steatosis and dietary antioxidant interventions, we analyzed the expression of three key genes: Nfe2l2 (Nrf2 pathway regulator), LpL, and FASN (Table 4). The observed transcriptional changes reflect both the progression of metabolic dysfunction and the therapeutic potential of antioxidant supplementation.

Nfe2l2 (Nuclear factor, erythroid 2-like 2), a key transcription factor to the Nrf2 antioxidant signaling pathway, showed marked upregulation in response to supplementation. The highest expression levels were observed in the CA group (2.90 ± 0.02) and the CAK group (2.83 ± 0.02), representing a significant increase compared to the control group C (1.00 ± 0.01; *p* < 0.001) (Table 4). These results confirm a strong activation of antioxidant response genes in animals supplemented with astaxanthin and the astaxanthin–kokum combination. In contrast, Nfe2l2 expression was markedly reduced in steatotic groups. The DA group showed the lowest expression (0.90 ± 0.02), while DAK showed a modest but significant restoration (1.13 ± 0.05), indicating partial reactivation of antioxidant signaling under oxidative stress. These findings reflect the suppressive impact of steatosis on cellular defense mechanisms, with antioxidant supplementation potentially reversing this trend.

Lipoprotein lipase expression, essential for triglyceride hydrolysis and lipid uptake, was significantly increased in group D (2.98 ± 0.08), compared to control group (1.01 ± 0.02), indicating impaired lipid clearance capacity. Antioxidants supplementation significantly restored LpL expression. The CAK group reached 0.64 ± 0.03, and DAK 0.49 ± 0.02, both significantly lower than in group D. These results suggest that combined astaxanthin and kokum supplementation can enhance lipid processing efficiency, especially under steatotic conditions.

Expression of FASN gene was markedly increased in steatotic groups, with values reaching 3.79 ± 0.17 in D and 2.16 ± 0.03 in DF (Table 4), consistent with active lipogenesis under lipid overload. In contrast, expression was drastically suppressed in all supplemented groups with the strongest reduction observed in the CAK (0.09 ± 0.00) and DAK (0.20 ± 0.02) groups. This indicates that the synergistic antioxidant intervention can effectively inhibit lipogenic gene programs even in metabolically stressed livers.

### Correlation

**Figure 9:**
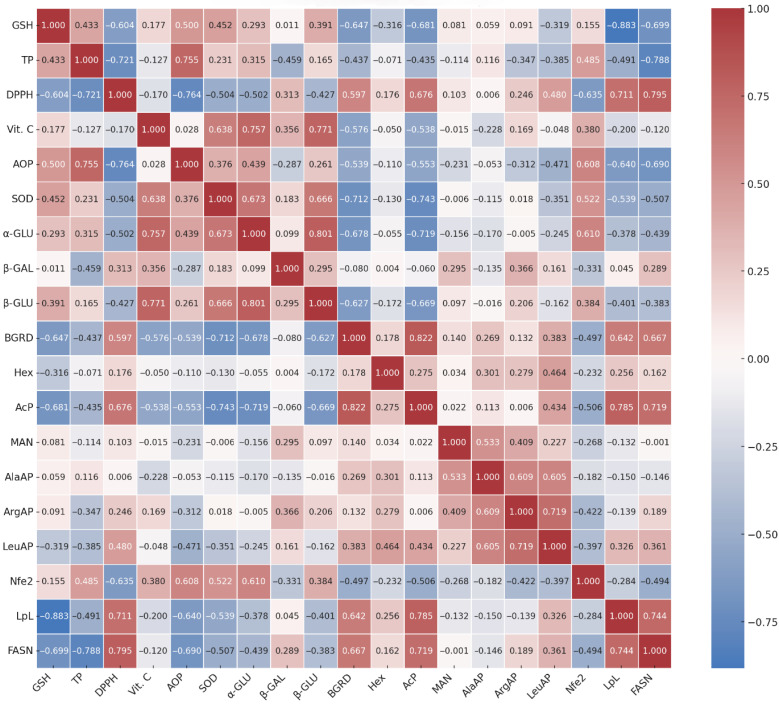
Pearson correlation heatmap illustrating associations among hepatic biomarkers across all experimental groups. The full correlation matrix is shown for completeness, but only the most biologically relevant associations are discussed in the main text.

To explore potential interactions between the evaluated biochemical, enzymatic, and gene expression markers, Pearson correlation coefficients (r) were calculated across all experimental groups (Figure 9). This analysis was exploratory and hypothesis-generating, and the observed correlations represent associations rather than causal effects. Given the comprehensive nature of the correlation matrix, we highlight below the associations most relevant to the study’s main conclusions.

Strong positive correlations were observed between GSH and key antioxidant markers, including total antioxidant capacity (r = 0.500) and polyphenol content (r = 0.433), confirming the synergistic action of endogenous and dietary antioxidant systems. In contrast, GSH showed a negative correlation with DPPH radical-scavenging capacity (r = −0.604), indicating that higher antioxidant reserves are associated with lower oxidative stress. A similar inverse association was observed between GSH and acid phosphatase (r = −0.681), a known lysosomal stress marker, suggesting that oxidative imbalance may contribute to enhanced catabolic lysosomal activation.

Polyphenols showed a robust positive correlation with AOP (r = 0.755) and a strong negative correlation with DPPH (r = −0.721), reinforcing their central role in maintaining hepatic redox homeostasis. These findings support the contribution of dietary phenolics to antioxidant capacity and their protective role under metabolic stress conditions.

DPPH was positively correlated with lysosomal stress markers: β-glucuronidase (r = 0.597) and AcP (r = 0.676) (Figure 9), consistent with a potential association between oxidative status and lysosomal activity, particularly in steatotic livers.

Vitamin C exhibited strong positive correlations with SOD (r = 0.638), α-glucosidase (r = 0.757), and β-glucosidase (r = 0.771), implicating its potential role in stabilizing redox-sensitive lysosomal enzymes. In contrast, AcP was negatively correlated with antioxidant markers, including GSH (r = −0.681), polyphenols (r = −0.435), and vitamin C (r = −0.538), supporting its utility as a biomarker of cellular injury and lysosomal stress.

High internal consistency was observed among lysosomal enzymes: α-glucosidase—β-glucosidase: r = 0.801. These enzymes also correlated positively with vitamin C (r = 0.757; 0.771, α-GLU; β-GLU, respectively), suggesting coordinated upregulation during adaptive or protective cellular responses. BGRD demonstrated a strong positive correlation with AcP (r = 0.822), reinforcing their joint involvement in catabolic activity during hepatic stress. Correlations involving additional enzymes, particularly aminopeptidases, were generally moderate, yet consistently observed: ArgAP-LeuAP, LeuAP-AlaAP, AlaAP-ArgAP, respectively, r = 0.719, 0.605, 0.609. These associations suggest coordinated regulation of proteolytic activity involved in extracellular matrix remodeling and immune signaling. Notably, LeuAP showed inverse correlations with GSH (r = −0.319) and AOP (r = −0.471), indicating that enhanced proteolysis may be linked to oxidative stress or redox imbalance.

Gene expression data revealed key correlations linking transcriptional regulation with oxidative and metabolic states. The Nrf2 pathway regulator correlated positively with: AOP (r = 0.608), polyphenols (r = 0.485), vitamin C (r = 0.380), SOD (r = 0.522), α-glucosidase (r = 0.610), and negatively with: FASN (r = −0.494), LpL (r = −0.284) (Figure 9), consistent with an association between promoting antioxidant defense and suppressing lipogenic responses under oxidative stress. Lipoprotein lipase expression was inversely correlated with GSH (r = −0.883), polyphenols (r = −0.491), and AOP (r = −0.640), and positively correlated with DPPH (r = 0.711), suggesting impaired lipid metabolism in redox-compromised states. Fatty acid synthase followed a parallel pattern, showing negative correlations with GSH (r = −0.699) and polyphenols (r = −0.788), and a strong positive correlation with DPPH (r = 0.795), suggesting a potential association in which lipogenesis is enhanced under oxidative pressure and may be attenuated through antioxidant intervention. Additionally, LpL and FASN showed a strong positive mutual correlation (r = 0.744), reflecting the coordinated activation of lipid uptake and synthesis during steatosis progression.

## 3. Discussion

Non-alcoholic fatty liver disease represents one of the most prevalent chronic metabolic disorders, encompassing a spectrum of liver abnormalities from simple steatosis to non-alcoholic steatohepatitis, progressive fibrosis, cirrhotic transformation, and hepatocellular carcinoma. Accumulating evidence highlights that oxidative stress is a central mechanism driving hepatocyte injury and disease progression [35]. Consistent with this concept, NAFLD groups in our study exhibited significant reductions in glutathione, vitamin C, and total antioxidant capacity, with reduced DPPH radical-scavenging capacity, reflecting depletion of redox defense systems.

This redox imbalance aligns with [14], who reported that decreased GSH:GSSG ratios correlate with NASH and fibrotic transformation. Under physiological conditions, this ratio is approximately 100:1 but can fall to 10:1 or even 1:1 under stress, triggering apoptotic and necrotic pathways. Furthermore, mitochondria are recognized as a major source of ROS in hepatocytes, with dysregulated β-oxidation in NAFLD enhancing oxidative damage [1,36]. Our data indirectly support mitochondrial dysfunction, as NAFLD groups exhibited elevated activities of lysosomal enzymes (AcP, BGRD, HEX), indicating increased catabolic and compensatory degradation of damaged proteins and organelles.

This is concordant with recent data from Pu [37], demonstrating that lysosomal dysfunction and elevated AcP activity are hallmarks of lipotoxicity and impaired autophagy in NAFLD. Additionally, Zeng et al. [38] showed that restoring lysosomal acidification reverses proteolytic dysfunction and ameliorates oxidative stress. In our study, supplementation with astaxanthin and kokum (CAK, DAK) significantly lowered AcP and BGRD activities, suggesting normalization of degradation pathways.

These enzymatic results are also supported by our gene expression analysis. Nfe2l2, a master regulator of antioxidant defenses, was strongly downregulated in NAFLD groups, consistent with studies by Mardinoglu et al. [19] and Qu et al. [39]. Supplementation restored Nfe2l2 expression, particularly in DAK, with parallel suppression of FASN, an essential enzyme involved in de novo lipogenesis. These effects may be linked to enhanced availability of GSH precursors such as cysteine and glycine [18] and modulation of upstream regulators such as miR-29a [7] or non-canonical NF-κB signaling [40].

Accordingly, GSH and SOD-core components of intracellular antioxidant defense-were both significantly depleted in NAFLD and restored by supplementation. These changes confirm earlier findings showing that GSH depletion and reduced SOD activity are hallmarks of NAFLD progression. Exogenous SOD3 (EC-SOD) has been shown to activate the AMPK-PGC1α axis and suppress inflammatory and lipogenic pathways [12], and co-supplementation with astaxanthin may enhance this effect [41].

Parallel improvements in polyphenols, AOP, and reduced DPPH, together with decreased AcP and BGRD activities, support the conclusion that astaxanthin and kokum exert potent antioxidant and cytoprotective effects. Notably, HEX decreased in steatotic mice receiving astaxanthin or kokum alone, whereas the combined steatotic group did not show a consistent decrease, indicating a non-additive interaction. This aligns with literature describing polyphenols and carotenoids as agents that reduce lipid peroxidation, enhance Nrf2 activity, inhibit lipogenesis, and activate β-oxidation [42].

At the enzymatic level, NAFLD was associated with increased lysosomal activity, reflected by elevated activities of AcP, BGRD, and HEX. These enzymes participate in the hydrolysis of glycoproteins and lipids and are known to be upregulated in liver injury. Following supplementation, the activities of these lysosomal markers were reduced, consistent with a partial normalization of degradative processes. Notably, AcP and BGRD decreased predominantly after combined supplementation, whereas HEX showed a more pronounced reduction under single-compound supplementation, indicating marker-specific and regimen-dependent lysosomal responses. Lysosomes, beyond their degradative role, act as metabolic signaling centers regulating nutrient sensing and gene expression. In NAFLD, lysosomal dysfunction—marked by elevated pH, reduced acid hydrolase activity, and impaired autophagosome-lysosome fusion—disrupts autophagic flux and aggravates lipotoxic stress [38].

In this context, the activity of aminopeptidases—LeuAP, ArgAP, and AlaAP—provided insights into protein catabolism and inflammation. Their modulation in response to dietary intervention supports their role as metabolic sensors. The reduced activity aminopeptidases in group D indicate impaired proteolytic capacity associated with lipid accumulation. However, supplementation—particularly when applied synergistically—restored or even enhanced ArgAP and AlaAP activity in the steatosis groups, suggesting a return to proteolytic homeostasis. A modest increase in the activity of the analyzed aminopeptidases was also observed after supplementation relative to the corresponding non-steatosis groups, indicating distinct proteolytic responses dependent on both disease status and the applied intervention.

Recent studies identified LAP3 (leucyl-aminopeptidase 3) as a potential biomarker of NASH, linked to degradation suppression and hepatic lipid accumulation [43,44]. In our study, LeuAP suppression by supplementation may reflect rescue of autophagic flux and proteostasis. Similar patterns were observed in proteomic analyses by Fourman et al. [45], linking aminopeptidase activation to fibrosis and immune responses in metabolic liver disease.

These interpretations are further informed by correlation analyses. Positive associations were observed between DPPH and AcP (r = 0.676), BGRD (r = 0.597), while negative correlations emerged between GSH and FASN (r = −0.699), and Nfe2l2 and LpL (r = −0.284), consistent with a potential inverse relationship between oxidative stress, lipid metabolism, and antioxidant defenses. The correlation of Nfe2l2 with AOP (r = 0.608), polyphenols (r = 0.485), and SOD (r = 0.522) may reflect its role as a central redox-sensitive transcriptional node.

Gene expression patterns of Nfe2l2, LpL, and FASN effectively distinguished NAFLD from healthy liver. Suppressed Nfe2l2, along with upregulated FASN and LpL, reflect oxidative stress, impaired lipid clearance, and enhanced lipogenesis in steatotic liver (D). Antioxidant supplementation, including the synergistic combination, reversed these alterations, likely contributing to the restoration of redox homeostasis and the improvement of lipid metabolism, thereby indicating a promising therapeutic potential.

Collectively, our data suggest that astaxanthin and *Garcinia indica* supplementation is associated with coordinated changes in the expression of key genes related to redox balance, lipid metabolism, and cellular degradation in the NAFLD model. These patterns are consistent with potential interactions among Nrf2-, inflammatory-, and nutrient-sensing pathways, although direct causal links cannot be inferred from the present dataset and should be explored in future targeted studies.

Simultaneously, the suppression of FASN expression, particularly in DAK group, indicates an inhibition of lipogenic activity, likely downstream of reduced oxidative and inflammatory signaling. The partial restoration of LpL expression may be indicative of enhanced lipid clearance and could suggest a potential attenuation of NF-κB-mediated inflammatory suppression.

Reductions in the activity of AcP and BGRD imply re-established degradative capacity, potentially linked to indirect modulation of TFEB, a known target of mTORC1. HEX activity was reduced mainly in steatotic mice receiving single-compound supplementation, suggesting that lysosomal remodeling may differ between mono- and co-supplementation. As FASN and LpL are involved in acetyl-CoA and nutrient flux, their transcriptional regulation may feed back into broader signaling networks affecting redox and catabolic balance [46].

Taken together, the observed changes in Nfe2l2, FASN, and LpL expression position these genes as functional nodes within a unified response to antioxidant intervention, linking the Nrf2, NF-κB, and mTOR/TFEB pathways in the restoration of hepatocellular homeostasis.

Finally, aminopeptidase activity (particularly LeuAP) showed inverse relationships with AOP and GSH, as noted by Runquist and Havel [47], indicating that oxidative depletion promotes protease activation. These enzymes may thus serve as early biomarkers of hepatocellular injury and potential therapeutic targets.

## 4. Materials and Methods

The study was conducted on 120 male Swiss Webster mice, 3 months of age, which were randomly allocated into two main groups: a control group (C; *n* = 60), receiving a standard laboratory diet, and an experimental group (D; *n* = 60), which was fed a modified diet intended to induce hepatic steatosis. Randomization was achieved through random selection of animals from the available population using a simple random draw method (equal probability), performed by an independent technician not involved in the downstream experimental procedures.

All animals were housed in the Animal Facility of the Institute of Genetics and Animal Biotechnology of the Polish Academy of Sciences (Jastrzębiec, Warsaw, Poland) under controlled environmental conditions: temperature of 22 ± 1 °C; 12:12 h light/dark cycle, and in conventional plastic cages where food and water were available ad libitum. All procedures involving animals were conducted in accordance with institutional ethical guidelines and the Directive 2010/63/EU on the protection of animals used for scientific purposes [48]. Ethical approval was granted by the Local Ethical Committee for Animal Experiments (approval no. WAW2/092/2024, 24 July 2024, II LEC in Warsaw).

**Figure 10:**
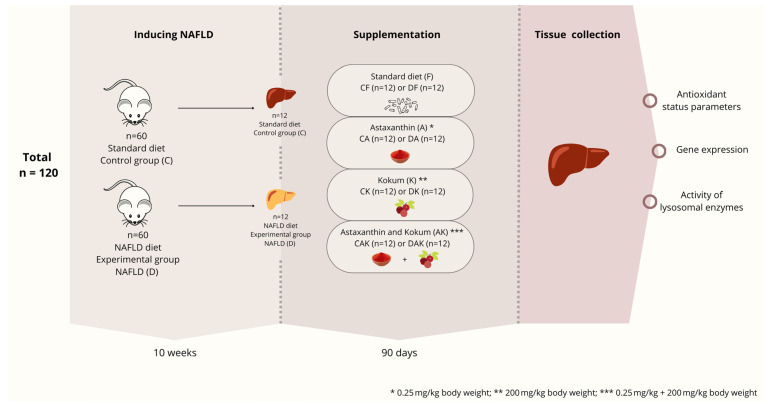
Experimental scheme of astaxanthin and kokum supplementation in a mouse model of NAFLD.

Initially, animals in the D group received a methionine- and choline-deficient (MCD) diet for a period of 10 weeks. Following this induction phase, both the C and D groups were each subdivided into four subgroups (*n* = 12 per subgroup), according to the supplementation scheme (Figure 10).

After the induction of hepatic steatosis, a 90-day supplementation period was initiated. All animals were fed a standard diet, which was modified according to group allocation as follows: no supplementation (CF and DF groups); supplementation with astaxanthin at a dose of 0.25 mg/kg body weight (CA and DA groups); supplementation with kokum at a dose of 200 mg/kg body weight (CK and DK groups); or combined supplementation with astaxanthin (0.25 mg/kg body weight) and kokum (200 mg/kg body weight), administered to the CAK and DAK groups. All animals had ad libitum access to the feed.

All supplements were administered in the form of dried powders. The kokum extract contained 20% garcinol (Livinol™, Sabinsa Corporation, East Windsor, NJ, USA), while the astaxanthin preparation (2.5% active compound) was supplied by Algamo s.r.o. (Mostek, Czech Republic). Supplemented feed was freshly prepared twice weekly by homogenously mixing the specified quantities of supplements into the standard laboratory chow. The mixtures were stored at room temperature until administration.

Throughout the study, mice were maintained in groups of four per cage. Animal welfare was monitored daily by a veterinarian. Weekly body weight measurements were performed by the same designated person (Table 5). Additionally, water and feed intake were monitored twice a week in each group. If signs of distress occurred, animals were to be subjected to a predefined humane endpoint via cervical dislocation.

At the end of the supplementation phase, all animals were euthanized by decapitation [49]. Liver tissues were immediately harvested, and processed for subsequent biochemical and histological analyses, as described in the following sections.

### 4.1. Measurement of Lysosomal Enzymes Activity

Liver homogenate samples were subjected to three freeze–thaw cycles prior to measuring lysosomal enzyme activities. Then, the samples were centrifuged at 4 °C (1500× *g* for 15 min) to obtain the supernatant. Activities of alanyl-aminopeptidase (AlaAP, EC 3.4.11.2), leucyl-aminopeptidase (LeuAP, EC 3.4.11.1), and arginyl-aminopeptidase (ArgAP, EC 3.4.11.6) were determined spectrophotometrically at 540 nm following a 1 h incubation at 37 °C, according to the method described by McDonald and Barrett [50]. The activities of acid phosphatase (AcP, EC 3.1.3.2), β-glucuronidase (BGRD, EC 3.2.1.31), α-glucosidase (α-GLU EC 3.2.1.20), β-glucosidase (β-GLU, EC 3.2.1.21), β-galactosidase (β-GAL, EC 3.2.1.23), N-acetyl-hexosaminidase (HEX), and mannosidase (MAN, EC 3.2.1.24) were measured as 4-nitrophenyl derivatives after incubation at 37 °C at 420 nm in accord with Barrett and Heath [51] method. Enzyme activity was measured using a UV-VIS CarryBio 50 spectrophotometer (Santa Clara, CA, USA) and expressed as nmol/mg protein/h.

### 4.2. Superoxide Dismutase (SOD E.C 1.15.1.1) Activity

Liver samples were homogenized in 5 mL of cold 20 mM HEPES buffer (pH 7.2) containing 1 nM EGTA, 210 mM mannitol, and 70 mM sucrose. The homogenates were centrifuged at 1500× *g* for 5 min at 4 °C, and the supernatants were kept on ice to prevent premature reaction initiation. SOD activity was assessed using the Superoxide Dismutase Assay Kit (Item No. 706002, Cayman Chemical, Ann Arbor, MI, USA). Absorbance was measured at 460 nm with a Synergy4 microplate reader (BioTek, Winooski, VT, USA), and enzyme activity was calculated using Gen5 software (version 3.15) (BioTek) and expressed in U/mL.

### 4.3. Reduced Glutathione Content

Glutathione levels were quantified with the OxisResearch™ Bioxytech^®^ GSH/GSSG-412™ assay (Foster City, CA, USA). For sample preparation, M2VP (1-methyl-2-vinyl-pyridinium trifluoromethanesulfonate) was added, and the material was stored at −80 °C until analysis. All procedures were carried out in accordance with the manufacturer’s protocol. Absorbance readings were obtained at 412 nm using a Synergy4 microplate reader, and data processing was performed with Gen5 software. The glutathione levels were calculated as μM concentration values.

### 4.4. Level of Vitamin C (Ascorbic Acid)

Liver tissue was homogenized in phosphate buffer (pH 7.0), after which 10% trichloroacetic acid was added. The mixtures were centrifuged at 3000× *g* for 10 min, and the resulting supernatants were combined with phosphoric acid (V), 2,2′-bipyridyl, and iron chloride (III). Samples were then incubated at 37 °C for 1 h. Absorbance was read at 525 nm using a UV-VIS CarryBio 50 spectrophotometer (Santa Clara, CA, USA). Standard curves were prepared with vitamin C solutions (0.5–5 mg; L-ascorbic acid, 99%, A92902, Sigma-Aldrich, St Louis, MO, USA) following the same procedure. Results were expressed as mg of vitamin C per 100 g of tissue.

### 4.5. Total Polyphenols Content

Liver tissue was homogenized in ultra-pure methanol containing 1% acetic acid, followed by a 2 h extraction in an ultrasonic bath at 40 °C. The samples were then centrifuged at 4000× *g* for 10 min at 4 °C. Total polyphenol content was determined using a modified version of the Škerget et al. [52] protocol, based on a colorimetric redox reaction. The resulting supernatant was transferred to 6 mL tubes and mixed with 2.5 mL of Folin–Ciocalteu reagent, diluted 10-fold with demineralized water (Sigma-Aldrich, St Louis, MO, USA). Sodium carbonate solution was added prior to incubation at 40 °C for 30 min. Absorbance was subsequently recorded at 765 nm and 735 nm. For the blank, 0.5 mL of ddH_2_O replaced the supernatant. Polyphenol concentration was calculated from a calibration curve generated with gallic acid standards and expressed as mg gallic acid equivalents (GAE) per g of tissue.

### 4.6. Free DPPH Radical Scavenging Potential

The antioxidative activity of the tested material was assessed following a modified version of the procedure described by Brand-Williams et al. [53], utilizing 1,1-diphenyl-2-picrylhydrazyl (DPPH) as a synthetic radical. Samples were homogenized in ultra-pure methanol with 1% acetic acid. Then, the homogenates were extracted for two hours in an ultrasonic bath at 40 °C. Afterwards, the tubes were centrifuged at 4000× *g* for 10 min at 4 °C. 0.5 mL of each obtained supernatant was collected and mixed with 0.5 mL of an ethanolic solution of 1,1-diphenyl-2-picrylhydrazyl (0.5 mM). Prior to addition, the solution was diluted to achieve an absorbance of approximately 0.9 at 517 nm. The samples were then incubated in a dark, cool environment for 30 min to allow color stabilization. Measurements were performed using a UV-VIS CarryBio 50 spectrophotometer (Santa Clara, CA, USA) at 517 nm.

### 4.7. Total Antioxidant Capacity Content

Using the Antioxidant Assay Kit (Item No. 709001, Cayman Chemical, Ann Arbor, MI, USA), TAC was measured based on the ABTS^®^ radical cation decolorization assay. The method measures the capacity of antioxidants in the samples to inhibit the oxidation of ABTS^®^ to ABTS^®^•+ by metmyoglobin in the presence of hydrogen peroxide [54]. Tissue homogenates were prepared in assay buffer (5 mM potassium phosphate, pH 7.4), centrifuged, and assayed in duplicate. Absorbance was recorded at 750 nm after a 5 min incubation at room temperature. A Trolox standard curve was plotted, and the antioxidant capacity of the samples was presented as millimolar (mM) Trolox equivalents (TE). The results were determined using the Gen5 software.

### 4.8. Histological Analysis

Liver fragments were promptly removed from mice immediately after decapitation. The samples were finely chopped and immersed in a fixative consisting of 2.5% glutaraldehyde in 0.1 M sodium cacodylate buffer (pH 7.2) for two hours at 4 °C. After primary fixation, tissue fragments were rinsed in the same buffer and subsequently post-fixed in a 1% osmium tetroxide solution prepared in 0.1 M cacodylate buffer for two hours at room temperature. The samples were washed with buffer and dehydrated through a graded ethanol series, with each step lasting 10 min. The dehydrated tissues were infiltrated and embedded in epoxy resin using propylene oxide as an intermediary solvent, following standard protocols. The embedded samples were polymerized in silicone moulds at 60 °C for 48 h, resulting in hardened resin blocks containing the preserved tissue.

Semi-thin sections (500 nm) were cut using a Reichert-Jung ultramicrotome (Reichert, Vienna, Austria), and then spotted on water droplets on glass slides, and dried on a hot plate at 60 °C to ensure adhesion. These sections were stained at 80 °C with Sudan III and aniline blue. After staining, the slides were thoroughly rinsed under running water, air-dried, mounted with Histokitt, and examined under a light microscope. Representative fields of each section were photographed using a Nikon Eclipse 80i microscope equipped with Nikon NIS Elements D 3.10 imaging software (Nikon Instruments, Inc., Melville, NY, USA).

### 4.9. RNA Extraction, Reverse Transcription, and Quantitative Real-Time PCR (qRT-PCR)

Total RNA was extracted from liver tissue using the RNeasy Mini Kit (Qiagen, Hilden, Germany) in accordance with the manufacturer’s guidelines. The RNA concentration and purity were assessed using a NanoDrop™ 2000 Spectrophotometer (Thermo Fisher Scientific, Waltham, MA, USA) by measuring absorbance at 230, 260, and 280 nm. Isolated RNA was maintained at −80 °C until further processing. Subsequently, cDNA was synthesized by reverse transcribing total RNA (1 μg) from each sample using the iScript™ cDNA Synthesis Kit (Bio-Rad Laboratories, Hercules, CA, USA), following the protocol provided by the manufacturer. The reverse transcription reaction (20 μL total volume) was conducted with the following thermal profile: 5 min at 25 °C, 20 min at 46 °C, and 1 min at 95 °C.

Quantitative real-time PCR (qRT-PCR) was performed on the CFX Opus 96 system (Bio-Rad, Richmond, CA, USA). Each 20 μL reaction mixture included 10 μL of 2X SsoAdvanced Universal SYBR Green Supermix (Bio-Rad), 500 nM of primers (Table 6), and 10 ng of cDNA. The qPCR cycling conditions were initial denaturation at 95 °C for 30 s, followed by 40 cycles consisting of 95 °C for 15 s and 60 °C for 30 s for annealing and extension. A Ct value greater than 37 was considered indicative of a negative result for target gene expression. A control negative sample, selected as a healthy mouse from the 3-month Control group, was used as the calibrator sample. Using GAPDH as the internal reference, relative expression levels of genes were determined via the 2-ΔΔCT method [55,56].

The selection of GAPDH was based on previous reports confirming its stable and consistent expression in murine liver tissue under comparable physiological and experimental conditions. For instance, Araujo et al. [57] demonstrated that GAPDH was one of the most stably expressed housekeeping genes in mouse hepatic tissue across varying experimental interventions. Similarly, Bruce et al. [58] highlighted the suitability of GAPDH for normalization purposes in hepatic gene expression studies, reinforcing its relevance and reliability as a reference gene.

Data were normalized and analyzed using CFX Maestro V2.2 software (Bio-Rad). Each gene expression measurement was performed in technical triplicates with biological replicates derived from independent cell cultures.

**Table 6:** Sequences of primers used in quantitative real-time PCR (qRT-PCR) analysis of gene expression.

Gene ^1^	Nucleotide Sequence (5′–3′)	Reference
FASN	F: GGAGGTGGTGATAGCCGGTAT R: TGGGTAATCCATAGAGCCCAG	Yang et al. [59]
LpL	F: GGGAGTTTGGCTCCAGAGTTT R: TGTGTCTTCAGGGGTCCTTAG	Huang et al. [60]
Nfe2l2	F: CCAGCACATCCAGACAGACAC R: GATATCCAGGGCAAGCGACTC	Chui et al. [61]
GAPDH	F: CTCCCACTCTTCCACCTTCG R: GCCTCTCTTGCTCAGTGTCC	He et al. [56]

^1^ FASN: Fatty Acid Synthase; LpL: Lipoprotein Lipase; Nfe2l2: Nuclear factor erythroid 2-related factor 2; GAPDH: Glyceraldehyde-3-Phosphate Dehydrogenase.

Data from qRT-PCR were analyzed through GraphPad Prism, version 9.0.0 (Boston, MA, USA).

### 4.10. Statistical Analysis

Before proceeding with the analysis, data distribution normality was assessed through D’Agostino-Pearson test. Differences in antioxidant and degradative parameters, as well as gene expression (with supplementation as the in-dependent factor), were assessed using one-way analysis of variance (ANOVA) among the experimental groups. In cases of statistically significant differences (*p* < 0.05), Tukey’s post hoc test or Bonferroni-Sidak’s test were applied to determine specific pairwise group differences. Data were presented as mean ± standard deviation, with *p*-value < 0.05 considered statistically significant.

Pearson correlation analysis was performed to evaluate linear relationships between biochemical parameters, with correlation strength classified as weak (|r| < 0.3), moderate (0.3 ≤ |r| < 0.7), or strong (|r| ≥ 0.7).

## 5. Conclusions

Our findings demonstrate that dietary supplementation with astaxanthin and kokum exerts potent hepatoprotective effects in a mice model of NAFLD by restoring redox balance, modulating lysosomal degradation, and reprogramming metabolic gene expression. Antioxidant intervention elevated hepatic glutathione and total antioxidant capacity, reduced lysosomal hydrolase activity, and improved vitamin C and polyphenol levels. At the molecular level, antioxidant treatment upregulated Nfe2l2 and downregulated FASN, while partially rescuing LpL expression-key regulators of antioxidant defense, lipogenesis, and lipid clearance, respectively. Correlation analysis revealed that redox parameters were linked to lysosomal and transcriptional markers, indicating coordinated changes among oxidative stress markers, lysosome enzymes, and lipid metabolism. These data support the hypothesis that targeting the glutathione–lysosomal degradation axis may represent an effective strategy for mitigating hepatic metabolic dysfunction. This study provides a proposal of support for future research into antioxidant-based therapies for NAFLD and related disorders.

## 6. Limitations and Future Directions

This study has several limitations. Gene expression changes were assessed by qRT-PCR without protein-level or activity-based validation and should therefore be interpreted with caution. In addition, the combined astaxanthin–kokum supplementation did not result in consistently additive effects across all analyzed parameters, which limits broad conclusions regarding synergistic interactions. NAFLD was induced using a MCD diet, which reliably induces hepatic steatosis and oxidative stress but does not fully reflect the metabolic features of human NAFLD, such as obesity and insulin resistance; therefore, the findings should be interpreted in the context of the applied experimental model.

Future studies may examine selected transcriptional findings at the protein and functional levels and further investigate the effects of astaxanthin and kokum using additional experimental models, dosing strategies, and time points.

## Figures and Tables

**Table 1:** The redox parameters in mice liver (GSH—the level of reduced glutathione, TP—the level of total polyphenols, DPPH—radical scavenger, Vit. C—the level of vitamin C, AOP—the level of total antioxidant potential, SOD—the activity of superoxide dismutase).

		C	D	CF	DF	CA	DA	CK	DK	CAK	DAK	F-Test	*p*-Value
GSH [µmol]	LSMean	701.91 ^A^	337.87 ^C^	784.33 ^A^	862.73 ^B^	776.19 ^A^	902.05 ^B^	825.18 ^B^	911.43 ^B^	812.68 ^B^	719.44 ^A^	55.56	*p* < 0.001
	±SD	142.63	33.09	51.17	12.63	26.31	49.12	17.35	33.35	38.26	63.63		
TP [mg GAE/g]	LSMean	5.67 ^A^	4.99 ^D^	6.05 ^A^	5.28 ^D^	6.54 ^B^	6.68 ^B^	7.25 ^B^	7.18 ^B^	7.93 ^C^	7.52 ^C^	37.65	*p* < 0.001
	±SD	0.34	0.39	0.35	0.56	0.52	0.21	0.30	0.31	0.69	0.37		
DPPH [%]	LSMean	65.72 ^A^	78.45 ^D^	63.15 ^A^	64.49 ^A^	52.75 ^B^	57.83 ^C^	52.48 ^B^	59.29 ^A^	48.48 ^B^	56.10 ^C^	30.89	*p* < 0.001
	±SD	3.08	4.38	4.87	3.71	1.89	6.63	3.22	2.93	4.27	4.09		
Vit. C [mg/g]	LSMean	5.19 ^A^	2.68 ^B^	5.18 ^A^	5.08 ^A^	4.60 ^A^	2.99 ^B^	4.40 ^A^	2.35 ^B^	5.10 ^A^	2.81 ^B^	42.84	*p* < 0.001
	±SD	0.37	0.63	0.68	0.65	0.46	0.50	0.30	0.25	0.24	0.45		
AOP [mM TE]	LSMean	4.83 ^A^	4.30 ^D^	5.96 ^C^	5.87 ^A^	7.84 ^B^	6.92 ^C^	7.76 ^B^	6.71 ^C^	8.16 ^B^	7.44 ^B^	29.04	*p* < 0.001
	±SD	0.51	0.70	0.84	0.40	0.61	0.41	0.51	0.66	1.01	0.54		
SOD [U/mL]	LSMean	5.47 ^A^	2.60 ^C^	5.90 ^A^	5.59 ^A^	6.26 ^A^	4.02 ^B^	5.17 ^A^	4.28 ^B^	5.85 ^A^	5.03 ^B^	19.65	*p* < 0.001
	±SD	0.28	0.69	0.54	0.67	0.43	1.34	0.53	0.18	0.68	0.53		

LSMean—Least Squares Mean; SD—Standard Deviation; Groups marked with different letters (e.g., ^A^ vs. ^B^) differ significantly (*p* < 0.05); C—initial control group; D—hepatic steatosis group; CF—continued control group; DF—post-steatosis group; CA—control group supplemented with astaxanthin; DA—hepatic steatosis group supplemented with astaxanthin; CK—control group supplemented with kokum; DK—hepatic steatosis group supplemented with kokum; CAK—control group with combined supplementation; DAK—hepatic steatosis group with combined supplementation.

**Table 2:** The activity of lysosomal enzymes (nmol/mg protein/h) in mice liver.

		C	D	CF	DF	CA	DA	CK	DK	CAK	DAK	F-Test	*p*-Value
	LSMean	244.64 ^A^	131.33 ^D^	226.60 ^A^	227.05 ^A^	234.67 ^A^	171.01 ^C^	256.00 ^A^	145.56 ^D^	295.59 ^B^	193.64 ^C^	54.03	*p* < 0.001
α-GLU	±SD	19.69	9.50	11.89	10.74	17.21	19.44	24.37	28.32	21.27	13.43		
	LSMean	268.49 ^A^	234.05 ^A^	274.78 ^C^	268.57 ^A^	229.30 ^A^	221.34 ^B^	267.88 ^A^	217.99 ^B^	190.22 ^B^	198.84 ^B^	11.00	*p* < 0.001
β-GAL	±SD	16.23	20.12	10.53	27.57	14.02	20.47	30.98	18.44	43.63	28.05		
	LSMean	78.52 ^A^	44.52 ^D^	67.97 ^B^	73.37 ^B^	65.40 ^B^	53.35 ^C^	77.25 ^A^	58.75 ^C^	82.09 ^A^	54.10 ^C^	51.67	*p* < 0.001
β-GLU	±SD	5.47	1.98	3.10	4.71	2.77	6.20	6.01	5.09	4.36	4.48		
	LSMean	185.10 ^A^	292.49 ^D^	118.69 ^B^	172.04 ^A^	153.03 ^A^	182.05 ^A^	148.06 ^A^	192.67 ^C^	124.16 ^B^	192.54 ^C^	37.41	*p* < 0.001
BGRD	±SD	6.15	48.99	13.70	9.92	16.86	13.58	23.56	17.01	10.51	18.18		
	LSMean	682.11 ^A^	808.45 ^B^	748.59 ^A^	783.80 ^B^	581.38 ^A^	613.26 ^A^	688.36 ^A^	601.44 ^A^	693.72 ^A^	973.06 ^B^	7.85	*p* < 0.001
HEX	±SD	36.91	178.72	44.40	23.74	47.29	105.01	89.47	28.69	48.44	250.99		
	LSMean	1556.65 ^A^	3002.79 ^C^	1530.33 ^A^	1535.61 ^A^	1292.73 ^A^	1829.97 ^B^	1354.24 ^A^	1740.74 ^B^	1318.60 ^A^	1784.71 ^B^	49.18	*p* < 0.001
AcP	±SD	269.54	127.90	68.68	183.34	194.34	198.43	215.05	160.64	196.52	193.74		
	LSMean	313.24 ^A^	240.32 ^B^	223.94 ^B^	258.09 ^A^	258.55 ^A^	259.98 ^A^	277.51 ^A^	282.08 ^A^	198.55 ^B^	251.41 ^A^	5.30	*p* < 0.001
MAN	±SD	21.87	62.34	36.41	6.12	23.50	44.74	31.22	46.31	35.18	25.05		

LSMean—Least Squares Mean; SD—Standard Deviation; Groups marked with different letters (e.g., ^A^ vs. ^B^) differ significantly (*p* < 0.05); C—initial control group; D—hepatic steatosis group; CF—continued control group; DF—post-steatosis group; CA—control group supplemented with astaxanthin; DA—hepatic steatosis group supplemented with astaxanthin; CK—control group supplemented with kokum; DK—hepatic steatosis group supplemented with kokum; CAK—control group with combined supplementation; DAK—hepatic steatosis group with combined supplementation.

**Table 3:** The activity of aminopeptidases (nmol/mg protein/h) in mice liver.

		C	D	CF	DF	CA	DA	CK	DK	CAK	DAK	F-Test	*p*-Value
	LSMean	318.91 ^A^	260.17 ^B^	207.85 ^B^	295.53 ^A^	254.69 ^B^	290.56 ^A^	242.84 ^B^	304.40 ^A^	269.59 ^B^	333.52 ^C^	10.45	*p* < 0.001
AlaAP	±SD	38.92	42.17	29.94	19.73	31.28	28.43	38.54	14.54	21.70	33.92		
	LSMean	348.24 ^A^	227.80 ^B^	209.97 ^B^	385.77 ^A^	217.23 ^B^	236.37 ^B^	229.94 ^B^	254.44 ^B,C^	199.32 ^B^	270.88 ^C^	19.83	*p* < 0.001
ArgAP	±SD	13.36	33.52	22.34	18.17	36.85	33.70	64.88	31.72	36.18	47.68		
	LSMean	421.51 ^A^	395.90 ^A^	314.57 ^B^	387.35 ^A^	283.99 ^B^	345.08 ^A^	265.28 ^B^	311.86 ^B^	294.78 ^B^	371.16 ^A^	6.69	*p* < 0.001
LeuAP	±SD	26.84	57.06	44.67	39.15	77.97	45.88	74.96	14.15	55.25	68.76		

LSMean—Least Squares Mean; SD—Standard Deviation; Groups marked with different letters (e.g., ^A^ vs. ^B^) differ significantly (*p* < 0.05); C—initial control group; D—hepatic steatosis group; CF—continued control group; DF—post-steatosis group; CA—control group supplemented with astaxanthin; DA—hepatic steatosis group supplemented with astaxanthin; CK—control group supplemented with kokum; DK—hepatic steatosis group supplemented with kokum; CAK—control group with combined supplementation; DAK—hepatic steatosis group with combined supplementation.

**Table 4:** Hepatic Expression Levels of Selected Genes in Mice.

		C	D	CF	DF	CA	DA	CK	DK	CAK	DAK	F-Test	*p*-Value
	LSMean	1.00 ^A^	0.40 ^F^	1.01 ^A^	0.35 ^F^	2.90 ^B^	0.90 ^E^	1.15 ^D^	0.51 ^G^	2.83 ^C^	1.13 ^D^	7677.25	*p* < 0.001
Nfe2 l2	±SD	0.01	0.01	0.03	0.04	0.02	0.02	0.04	0.02	0.02	0.05		
	LSMean	1.01 ^A^	2.98 ^G^	1.00 ^A^	0.09 ^B^	0.09 ^B^	0.09 ^B^	0.22 ^D^	0.29 ^F^	0.64 ^C^	0.49 ^E^	6066.18	*p* < 0.001
LpL	±SD	0.02	0.08	0.02	0.01	0.00	0.00	0.02	0.01	0.03	0.02		
	LSMean	1.05 ^A^	3.79 ^G^	1.00 ^A^	2.16 ^F^	0.88 ^B^	0.65 ^D^	0.81 ^B^	0.58 ^D^	0.09 ^C^	0.20 ^E^	2401.15	*p* < 0.001
FASN	±SD	0.05	0.17	0.01	0.03	0.01	0.03	0.01	0.02	0.00	0.02		

LSMean—Least Squares Mean; SD—Standard Deviation; Groups marked with different letters (e.g., ^A^ vs. ^B^) differ significantly (*p* < 0.05); C—initial control group; D—hepatic steatosis group; CF—continued control group; DF—post-steatosis group; CA—control group supplemented with astaxanthin; DA—hepatic steatosis group supplemented with astaxanthin; CK—control group supplemented with kokum; DK—hepatic steatosis group supplemented with kokum; CAK—control group with combined supplementation; DAK—hepatic steatosis group with combined supplementation.

**Table 5:** Body weight of mice fed the MCD or control diet for 10 weeks.

Parameter	Control Diet	MCD Diet
Baseline body weight (g)	30.3 ± 0.5	29.9 ± 0.4
Final body weight (g)	33.8 ± 0.6	32.8 ± 0.8
Change in body weight (g)	3.4 ± 0.5	2.9 ± 0.6
(%)	(11.6%)	(9.7%)

Values represent means ± SEM; *n* = 60 for each group.

## Data Availability

The data used and/or analyzed in this investigation are available from the corresponding authors upon reasonable request.
